# Prioritizing potential circRNA biomarkers for bladder cancer and bladder urothelial cancer based on an ensemble model

**DOI:** 10.3389/fgene.2022.1001608

**Published:** 2022-09-15

**Authors:** Qiongli Su, Qiuhong Tan, Xin Liu, Ling Wu

**Affiliations:** Department of Pharmacy, The Affiliated Zhuzhou Hospital Xiangya Medical College CSU, Zhuzhou, Hunan, China

**Keywords:** bladder cancer, bladder urothelial cancer, circRNA, biomarker, circRNA-disease association, ensemble learning

## Abstract

Bladder cancer is the most common cancer of the urinary system. Bladder urothelial cancer accounts for 90% of bladder cancer. These two cancers have high morbidity and mortality rates worldwide. The identification of biomarkers for bladder cancer and bladder urothelial cancer helps in their diagnosis and treatment. circRNAs are considered oncogenes or tumor suppressors in cancers, and they play important roles in the occurrence and development of cancers. In this manuscript, we developed an Ensemble model, CDA-EnRWLRLS, to predict circRNA-Disease Associations (CDA) combining Random Walk with restart and Laplacian Regularized Least Squares, and further screen potential biomarkers for bladder cancer and bladder urothelial cancer. First, we compute disease similarity by combining the semantic similarity and association profile similarity of diseases and circRNA similarity by combining the functional similarity and association profile similarity of circRNAs. Second, we score each circRNA-disease pair by random walk with restart and Laplacian regularized least squares, respectively. Third, circRNA-disease association scores from these models are integrated to obtain the final CDAs by the soft voting approach. Finally, we use CDA-EnRWLRLS to screen potential circRNA biomarkers for bladder cancer and bladder urothelial cancer. CDA-EnRWLRLS is compared to three classical CDA prediction methods (CD-LNLP, DWNN-RLS, and KATZHCDA) and two individual models (CDA-RWR and CDA-LRLS), and obtains better AUC of 0.8654. We predict that circHIPK3 has the highest association with bladder cancer and may be its potential biomarker. In addition, circSMARCA5 has the highest association with bladder urothelial cancer and may be its possible biomarker.

## 1 Introduction

Bladder cancer is considered to be the most common cancer in the urinary system (Kamat et al., 2016). It is the fourth most common malignant tumor in men and the eighth most common in women in the Western world. In the United States and Europe, it accounts for 5%–10% among all malignancies in men. The risk with the bladder cancer infection at less than 75 years is 2%–4% in men and 0.5%–1% for women (Kirkali et al., 2005). The incidence of bladder cancer has been increasing (Kamat et al., 2016). The majority of patients with bladder cancer suffer from the less aggressive non-muscle-invasive disease, while 30% of patients suffer from muscle-invasive disease (Lopez-Beltran et al., 2021; Tran et al., 2021; Yang et al., 2021).

Bladder cancer has a metastatic potential, and thus presents a worse prognosis. It is usually grouped into three pathological categories: bladder urothelial carcinoma, bladder squamous cell carcinoma, and bladder adenocarcinoma (Black and Black, 2020; Lopez-Beltran et al., 2021). Bladder urothelial carcinoma accounts for over 90% among all cases of bladder cancer. Furthermore, bladder urothelial carcinoma can be categorized into muscle-invasive bladder cancer, which accounts for about 75% of all cases, and non-muscle-invasive bladder cancer (Kirkali et al., 2005). The all-stage five-year survival rate of bladder urothelial cancer remains approximately 80% (Lopez-Beltran et al., 2021).

Recently, the treatment of bladder cancer has obtained great progresses worldwide. Besides traditional surgical resection, radiotherapy, and chemotherapy, immunotherapy is also a promising avenue for bladder cancer treatment (Gao et al., 2021; Mancini et al., 2021). However, postoperative recurrence and distant metastasis cause five-year survival rates to still be very low for advanced bladder cancer (Fabiano et al., 2021; Roviello et al., 2021). Advanced disease or relapse of radical cystectomy is closely associated with the poor outcomes (Nouhaud et al., 2021). The first-line therapy of metastatic bladder urothelial cancer usually adopts cisplatin-based combinations, and has been unaltered over the last decades (Powles et al., 2021; Renner et al., 2021; Walia et al., 2021). Unfortunately, almost all patients with bladder urothelial cancer will finally progress and die from bladder cancer, despite their initial response to cisplatin-based combinations (Bin Riaz et al., 2021; Lopez-Beltran et al., 2021). Consequently, inferring potential biomarkers for bladder cancer is a good way to diagnose and treat it (Peng et al., 2017; Peng et al., 2018).

With the advance of sequencing technology, there are now massive amounts of RNA data (Ozsolak and Milos, 2011; Peng et al., 2020; Yang et al., 2020; Peng et al., 2022a), which help the prognosis and treatment of various diseases (Xu et al., 2020; Li et al., 2021). Circular RNAs (circRNAs) are a class of single-stranded noncoding RNA molecules that are lack of terminal 5′ caps and 3′ poly(A) tails (He et al., 2017). circRNAs are widely distributed in various organisms. They have circular features, and thus demonstrate more resistance to degradation by exonucleases and stronger stability than linear RNAs (Xia et al., 2018; Li G. et al., 2020). The estimated total number of circRNAs is approximately 1% of one of poly (A) molecules. In addition, the expression levels of the majority of circRNAs are estimated to be 5%–10% of the corresponding linear RNAs (Jeck and Sharpless, 2014; Zhang J. et al., 2021).

Although circRNAs were found in 1976, they were originally considered to be functionless by-products from aberrant RNA splicing and thus did not obtain enough attention over the past 3 decades. However, with the rapid advance of high-throughput sequencing technologies, massive differentially expressed circRNAs have been increasing discovered in human normal and malignant cells (Zhang et al., 2018; Li G. et al., 2020; Yang et al., 2021). circRNAs exist widely in various tissues, serum, and urine. The expression profiles of circRNAs demonstrate strong specificity in cell types, tissues, and developmental stages (Yang et al., 2021). Furthermore, circRNAs can regulate transcription or splicing, translate proteins, interact with RNA-binding proteins, and act as miRNA sponges (Sheng et al., 2018). A large body of evidence shows that circRNAs have dense associations with various diseases, including neurological dysfunction, cardiovascular diseases, and cancer. Here, circRNAs, as miRNA sponges, can inhibit the regulation from downstream cancer target genes. For instance, circCDR1as and circMTO1 can control gene regulation and further indirectly stimulate or inhibit tumors by binding to miR-7 and miR-9 (Vromman et al., 2021).

circRNAs have abundant associations with cancers and thus can be used as candidate cancer biomarkers (Zhang et al., 2018). An increasing amount of evidence has reported that circRNAs present in human biofluids and exosomes, and are a class of potential biomarkers of noninvasive liquid biopsies. For instance, circ-ZEB1.33 is overexpressed in hepatocellular cancer and has close links with the survival of hepatocellular cancer patients (Gong et al., 2018). In particular, substantial studies have demonstrated that circRNAs play key roles in the carcinogenesis and progression of bladder cancer. For example, circRNAs Cdr1as performs anti-oncogenic functions in bladder cancer through microRNA 135a (Li et al., 2018), BCRC-3 suppresses bladder cancer proliferation via sponging miR-182-5p/p27 (Xie et al., 2018), MYLK and circPDSS1 promote bladder cancer progression separately by modulating VEGFA/VEGFR2 signaling pathway and down-regulating miR-16 (Zhong et al., 2017; Yu et al., 2020), PRMT5 supports metastasis of bladder urothelial cancer through Sponging miR-30c (Chen et al., 2018), circSLC8A1 suppresses bladder cancer progression through regulating PTEN (Lu et al., 2019), and circMTO1 inhibits bladder cancer metastasis through sponging miR-221 (Li G. et al., 2019).

Many computational methods have been proposed to identify possible CDAs and further discovered possible circRNA biomarkers for various complex diseases including cancers by case studies (Wang CC. et al., 2021). For example, Lei et al. (Lei et al., 2018) designed a path weighted-based CDA prediction approach (PWCDA). Li et al. (Li Y. et al., 2019; Li J. et al., 2020) explored two CDA identification models (NCPCDA and DWNCPCDA) based on network consistency projection. Zhang et al. (Zhang et al., 2019) developed a linear neighborhood label propagation algorithm for CDA identification. Deepthi et al. (Deepthi and Jereesh (2020) used autoencoder and deep neural network and explored an ensemble model to predict CDAs. Lu et al. (Lu et al. (2021) improved CDA prediction using convolutional and recurrent neural networks. Wang et al. (Wang et al., 2020; Wang et al. 2021b; Wang et al., 2021c) proposed three CDA identification methods (GCNCDA, MGRCDA, and SGANRDA) based on graph convolutional network, metagraph recommendation, and semi-supervised generative adversarial network, respectively. These methods efficiently predicted possible CDAs.

In this study, inspired by computational CDA prediction methods, we develop an ensemble model, CDA-RWLRLS, to find potential circRNA biomarkers for bladder cancer and bladder urothelial cancer based on known CDAs. CDA-EnRWLRLS first computes circRNA similarity by integrating their functional similarity and association profile similarity, and it computes disease similarity by integrating their semantic similarity and association profile similarity. Second, CDA-EnRWLRLS computes the association probability for each circRNA-disease pair based on random walk with restart and Laplacian regularized least squares. Third, the prediction results obtained by these two models are integrated by the soft voting method. We finally use the proposed CDA-EnRWLRLS model to identify possible circRNAs associated with bladder cancer and bladder urothelial cancer.

## 2 Materials and methods

### 2.1 Materials

#### 2.1.1 Human circRNA-disease associations

circRNA-disease association data can be downloaded from the circR2Disease database (Fan et al., 2018a). This database provides 739 experimentally confirmed CDAs from 661 circRNAs and 100 diseases. We remove redundant elements related to mice and rats and achieve a human circRNA-disease association dataset containing 650 associations between 585 circRNAs and 88 diseases. In particular, suppose that $C=\left\{c_{1},\;c_{2},\ldots ,{\;c}_{m}\right\}$ and $D=\left\{d_{1},d_{2},\ldots ,d_{n}\right\}$ separately denote the sets of m circRNAs and n diseases, then we construct a binary matrix $Y\epsilon R^{m\times n}$ to depict circRNA-disease associations by Eq. 1:

$$Y_{ij}=\left\{ \begin{array}{c} 1\;If\;circRNA\;c_{i}\;associates\;with\;d_{j}\\ 0\;\;\;\;\;otherwise \end{array}\right.$$ (1)

#### 2.1.2 Disease semantic similarity

Many studies have computed disease semantic similarity to screen credible noncoding RNAs for a query disease. Inspired by these methods, we investigate disease similarity to improve the prediction performance. Disease semantic similarity can be computed based on corresponding disease ontology. The disease ontology is often represented using a directed acyclic graph and can be downloaded from http://disease-ontology.org/. For two query diseases and corresponding ontology term sets from the two diseases $d_{i}$ and $d_{j}$ , their semantic similarity can be scored by the “doSim” function in the DOSE software package, which can be downloaded from http://www.bioconductor.org/packages/release/bioc/html/DOSE.html (Yu et al., 2015). Finally, we compute the semantic similarity matrix $S_{d}^{sem}$ among n diseases.

#### 2.1.3 circRNA functional similarity

To measure the functional similarity between two circRNAs, we utilize the semantic similarity of two diseases linking to the two circRNAs. In particular, suppose that $D_{i}$ and $D_{j}$ denote the disease groups linking to circRNAs $c_{i}$ and $c_{j}$ , the functional similarity between $c_{i}$ and $c_{j}$ can be computed by Eq. 2:

$$S_{c}^{fun}=\frac{\underset{1\leq p\leq \left|D_{i}\right|}{\sum }S\left(d_{p},D_{j}\right)+\underset{1\leq p\leq \left|D_{j}\right|}{\sum }S\left(d_{q},D_{i}\right)}{\left|D_{i}\right|+\left|D_{j}\right|}$$ (2)

and

$$S\left(d_{p},D_{j}\right)=\max_{1\leq t\leq \left|D_{j}\right|}\left(S_{d}^{sem}\left(d_{p},d_{t}\right)\right)$$ (3)

where $S\left(d_{p},D_{j}\right)$ denotes the similarity between disease $d_{p}$ linking to circRNA $c_{i}$ and disease set $D_{j}$ linking to circRNA $c_{j}$ .

### 2.2 Methods

**FIGURE 1:**
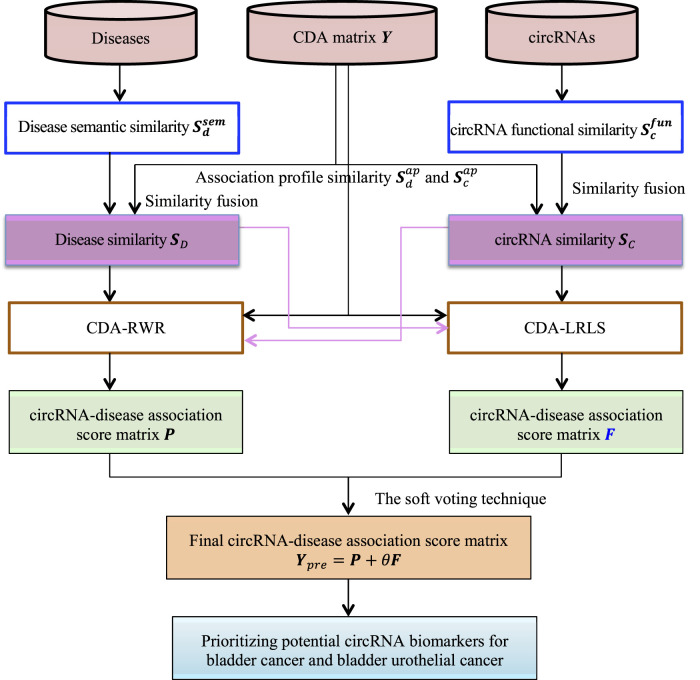
Flowchart of the proposed CDA-EnRWLRLS model.

In this manuscript, we develop circRNA-Disease Association prioritization method (CDA-EnRWLRLS) by an Ensemble of Random Walk with restart and Laplacian Regularization Least Squares. First, CDA-EnRWLRLS measures circRNA functional similarity and disease semantic similarity. Second, it computes association profile similarity of circRNAs and diseases, respectively. Third, functional similarity and association profile similarity of circRNAs are combined to obtain the final circRNA similarity. Similarly, disease similarity is fused. Fourth, random walk with restart and Laplacian regularization least squares are used to score each circRNA-disease pair. Fifth, the final association score matrix is obtained by integrating the results from random walk with restart and Laplacian regularization least squares based on the soft voting strategy. Finally, CDA-EnRWLRLS is applied to find possible circRNA biomarkers for bladder cancer and bladder urothelial cancer. The flowchart of CDA-EnRWLRLS is shown in Figure 1.

#### 2.2.1 Association profile similarity of circRNAs and diseases

For two diseases with known ontology terms, we can compute their semantic similarity based on their ontology terms. However, semantic similarity computation may fail for two diseases without ontology terms. Thus, we introduce association profile similarity to further complement similarity measurement of circRNAs and diseases.

Suppose that the association profile Y(i,:) of a circRNA $c_{i}$ is represented as the i th row of a CDA matrix Y . Y(i,:) describes information from all diseases associated with $c_{i}$ . Association profile similarity between two circRNAs (i.e., $\left(c_{i},c_{j}\right)$ ) can be computed by Eq. 4:

$$\begin{array}{c} S_{c}^{ap}\left(c_{i},c_{j}\right)=\exp\left({-\gamma_{c}\left\|Y\left(i,:\right)-Y\left(j,:\right)\right\|}^{2}\right)\\ \gamma_{c}=\gamma_{c}^{\prime }/\left(\frac{1}{m}\overset{m}{\underset{k=1}{\sum }}{\left\|Y\left(k,:\right)\right\|}^{2}\right) \end{array}\left(4\right)$$

where $\gamma_{c}^{\prime }$ is bandwidth parameter and set as the default value of 1.

Similarly, association profile similarity between two diseases (i.e., $\left(d_{i},d_{j}\right)$ ) can be computed by Eq. 5:

$$\begin{array}{c} S_{d}^{ap}\left(d_{i},d_{j}\right)=\exp\left({-\gamma_{d}\left\|Y\left(:,i\right)-Y\left(:,j\right)\right\|}^{2}\right)\\ \gamma_{d}=\gamma_{d}^{\prime }/\left(\frac{1}{n}\overset{n}{\underset{k=1}{\sum }}{\left\|Y\left(:,k\right)\right\|}^{2}\right) \end{array}\left(5\right)$$

where $\gamma_{d}^{\prime }$ indicates bandwidth parameter and set as the default value of 1.

#### 2.2.2 Similarity fusion

circRNA functional similarity $S_{c}^{fun}$ , disease semantic similarity $S_{d}^{sem}$ , and association profile similarity of circRNAs and diseases ( $S_{c}^{ap}$ and $S_{d}^{ap}$ ) are fused to obtain the final circRNA similarity matrix $S_{C}$ and disease similarity $S_{D}$ by Eqs 6, 7:

$$S_{C}=\alpha_{c}S_{c}^{fun}+\left(1-\alpha_{c}\right)S_{c}^{ap}$$ (6)

$$S_{D}=\alpha_{d}S_{d}^{sem}+\left(1-\alpha_{d}\right)S_{d}^{ap}$$ (7)

The parameter $\alpha_{c}$ is used to balance the importance between functional similarity and association profile similarity of circRNAs in Eq. 6 and $\alpha_{d}$ is used to balance the important between semantic similarity and association profile similarity of diseases in Eq. 7.

#### 2.2.3 Random walk with restart for CDA prediction

Random walk algorithm has been widely used and obtained better performance in various association prediction fields (Peng et al., 2021a). In this study, we utilize Random Walk with Restart for CDA prediction on the heterogeneous circRNA-disease network (CDA-RWR). We first train the random walk with restart model on the CDA dataset and screen possible CDAs with the highest association probability from unknown circRNA-disease pairs on the dataset.

First, circRNA similarity network $N_{c}$ , disease similarity network $N_{d}$ , and CDA network $N_{a}$ are used to build a heterogeneous circRNA-disease network. $S_{c}$ , $S_{d}$ , and Y correspond to adjacency matrices of the three networks, respectively. Consequently, the heterogeneous circRNA-disease network can be represented as: $W=\left[\begin{array}{cc} S_{C} & Y\\ Y^{T} & S_{D} \end{array}\right]$ , where $Y^{T}$ is the transpose of Y .

Second, we compute the transition probability of random walk on the heterogeneous circRNA-disease network. Suppose that $W=\left[\begin{array}{cc} W_{cc} & W_{cd}\\ W_{dc} & W_{dd} \end{array}\right]$ denote the transition matrix, where $W_{cc}$ and $W_{dd}$ separately indicate the walk within the circRNA network and the disease network, $W_{cd}$ and $W_{dc}$ separately represent the jump from the circRNA network to the disease network and the disease network to the circRNA network. For a known jumping probability μ from the circRNA network to the disease network or from the disease network to the circRNA network, the transition probability from circRNAs $c_{i}$ to $c_{j}$ can be calculated by Eq. 8:

$$W_{cc}\left(i,j\right)=\left\{ \begin{array}{ll} \frac{S_{C}\left(i,j\right)}{\overset{m}{\underset{k=1}{\sum }}S_{C}\left(i,k\right)} & if\;\overset{n}{\underset{k=1}{\sum }}Y\left(i,k\right)=0\\ \frac{\left(1-\mu \right)S_{C}\left(i,j\right)}{\overset{m}{\underset{k=1}{\sum }}S_{C}\left(i,k\right)} & otherwise \end{array}\right.,$$ (8)

The transition probability from circRNA $c_{i}$ to disease $d_{j}$ can be calculated by Eq. 9:

$$W_{cd}\left(i,j\right)=\left\{ \begin{array}{ll} \frac{\mu Y\left(i,j\right)}{\overset{n}{\underset{k=1}{\sum }}Y\left(i,k\right)} & if\;\overset{n}{\underset{k=1}{\sum }}Y\left(i,k\right)\neq 0\\ 0 & otherwise \end{array}\right.,$$ (9)

The transition probability from diseases $d_{i}$ to $d_{j}$ can be calculated by Eq. 10:

$$W_{dd}\left(i,j\right)=\left\{ \begin{array}{ll} \frac{S_{d}\left(i,j\right)}{\overset{n}{\underset{k=1}{\sum }}S_{d}\left(i,k\right)} & if\;\overset{m}{\underset{k=1}{\sum }}Y\left(k,i\right)=0\\ \frac{\left(1-\mu \right)S_{d}\left(i,j\right)}{\overset{n}{\underset{k=1}{\sum }}S_{d}\left(i,k\right)} & otherwise \end{array}\right.,$$ (10)

The transition probability from disease $d_{i}$ to circRNA $c_{j}$ can be calculated by Eq. 11:

$$W_{dv}\left(i,j\right)=\left\{ \begin{array}{ll} \frac{\mu Y\left(j,i\right)}{\overset{n}{\underset{k=1}{\sum }}Y\left(k,i\right)} & \;if\;\overset{n}{\underset{k=1}{\sum }}Y\left(k,i\right)\neq 0\\ 0 & \;otherwise \end{array}\right.,$$ (11)

For a query circRNA/disease, it can either stay in the current network with a restart probability β∈(0,1) or jump to another network graph. Consequently, we can compute association probability for each circRNA-disease pair at the (t+1) -th step by Eq. 12:

$$p_{t+1}=\beta Wp_{t}+\left(1-\beta \right)p_{0},$$ (12)

where $p_{t}$ denotes the association probability matrix at the t -th step, $p_{0}$ denotes the initial probability and $p_{0}=\left[\begin{array}{c} \lambda u_{0}\\ \left(1-\lambda \right)v_{0} \end{array}\right]$ , where $u_{0}$ and $v_{0}$ indicate the initial probability on the circRNA and disease network, respectively. When we want to discover possible circRNAs associated with a query disease $d_{i}$ , it is regarded as a seed in the disease network. Consequently, $d_{i}$ is assigned as 1 and other disease nodes are 0, thereby building the initial probability matrix of the disease network $v_{0}$ . All nodes in the circRNA network $u_{0}$ are assigned as an equal probability whose sum is 1. The parameter β is used to balance the importance of the circRNA network and the disease network.

### 2.3 Laplacian regularized least squares for CDA prediction

We can calculate association probability for each circRNA-disease pair based on random walk with restart. However, for random walk with restart, the jump probability is measured by known CDAs and the circRNA and disease similarity matrices. For a circRNA $c_{i}$ in a CDA network, if two other circRNAs $c_{j}$ and $c_{k}$ have the equal similarity with $c_{i}$ , $c_{j}$ and $c_{k}$ will contribute to the jump between nodes at an equal probability. However, the circRNA that exhibits lower similarities with other circRNAs should have more contribution to the jump. Thus, we further use Laplacian regularized least squares (Shen et al., 2022) to compute association probability for each circRNA-disease pair.

First, we compute the circRNA Laplacian matrix $L_{c}$ and the disease Laplacian matrix $L_{d}$ by Eqs 13, 14:

$$L_{c}={\left(A_{c}\right)}^{-1/2}\left(A_{c}-A_{c}\right){\left(A_{c}\right)}^{-1/2}$$ (13)

$$L_{d}{=\left(A_{d}\right)}^{-1/2}\left(A_{d}-A_{d}\right){\left(A_{d}\right)}^{-1/2}$$ (14)

where $A_{c}$ */* $A_{d}$ indicates the diagonal matrix of circRNA/disease similarity matrix and $A_{c}\left(i,i\right)$ */* $A_{d}\left(j,j\right)$ is the summation of the i -th/ j -th row of $S_{C}$ */* $S_{D}$ .

Second, we define the loss functions of Laplacian regularization least squares in the circRNA and disease spaces based on the Laplacian matrices $L_{c}$ and $L_{d}$ by Eqs 15, 16, respectively:

$$\min_{F_{c}}\left[{\left\|Y^{T}-F_{c}\right\|}_{F}^{2}+\gamma_{c}{\left\|F_{c}∙L_{c}∙{\left(F_{c}\right)}^{T}\right\|}_{F}^{2}\right]$$ (15)

$$\min_{F_{d}}\left[{\left\|Y-F_{d}\right\|}_{F}^{2}+\gamma_{d}{\left\|F_{d}∙L_{d}∙{\left(F_{d}\right)}^{T}\right\|}_{F}^{2}\right]$$ (16)

where $Y^{T}$ , ${\left(F_{c}\right)}^{T}$ , and ${\left(F_{d}\right)}^{T}$ separately indicate the transposes of Y , $F_{c}$ , and $F_{d}$ , ${\left\|∙\right\|}_{F}$ indicates the Frobenius norm, and $\gamma_{c}$ and $\gamma_{d}$ indicate trade-off parameters. The Laplacian regularized least square models (15) and (16) can be solved by Eqs 17, 18:

$$F_{c}=S_{C}{\left(S_{C}+\gamma_{c}∙L_{c}∙S_{c}\right)}^{-1}Y^{T}$$ (17)

$$F_{d}=S_{d}{\left(S_{d}+\gamma_{d}∙L_{d}∙S_{d}\right)}^{-1}Y$$ (18)

Finally, the association probability for each circRNA-disease pair by Laplacian regularized least squares can be computed by Eq. 19:

$$F=\frac{1}{2}\left(F_{c}+F_{d}\right)$$ (19)

### 2.4 Ensemble learning for CDA prediction

Ensemble learning integrates multiple results from individual models and demonstrates better performance compared to individual models (Zhou et al., 2021a; Peng et al., 2022b). Therefore, in this study, we develop an ensemble learning model by combining random walk with restart and Laplacian regularized least squares to improve the CDA’s prediction performance by Eq. 20:

$$Y_{pre}=P+\theta F$$ (20)

where $Y_{pre}$ denotes the predicted final CDA score matrix, P and F denote the computed CDA probability matrices based on random walk with restart and Laplacian regularized least squares, respectively. θ is used to weigh the importance of results computed by the above two models.

## 3 Experiments

### 3.1 Experimental settings

For similarity computation, the weights between biological feature similarity and association profile similarity $\alpha_{c}$ and $\alpha_{d}$ are set as 0.5. For random walk with restart, the restart probability β is set as 0.2, and λ and μ are set as 0.1 and 0.6, respectively. For Laplacian regularized least squares, both $\gamma_{c}$ and $\gamma_{d}$ are set as 0.95 and 0.2, respectively. For ensemble learning model, θ is set as 0.3. The parameters in other three comparative methods are set as defaults provided by the corresponding methods. We conduct 5-fold cross validation for 10 times. The final prediction performance is from the average value of the 10 experiments. AUC (area under the receiver operating characteristic curve) has been widely used to evaluate the performance of CDA prediction methods. Larger AUC denotes better performance. Thus, we use AUC to measure the performance of our proposed method.

### 3.2 Performance comparison with five CDA prediction methods

**FIGURE 2:**
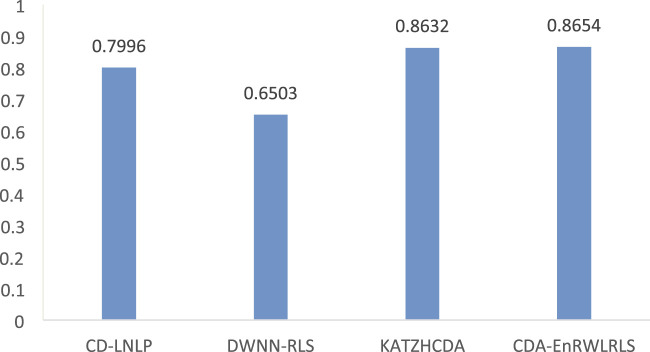
The AUC values of CDA-EnRWLRLS and other three method.

Several comparative experiments are conducted to measure the performance of our proposed CDA-EnRWLRLS model. CD-LNLP (Zhang et al., 2019), DWNN-RLS (Yan et al., 2018), KATZHCDA (Fan et al., 2018b), and CDA-EnRWLRLS are conducted on the preprocessed CDA dataset. CD-LNLP (Zhang et al., 2019) is a linear neighborhood label propagation-based algorithm for CDA prediction. DWNN-RLS (Yan et al., 2018) used regularized least squares to predict possible CDAs. KATZHCDA (Fan et al., 2018b) discovered CDA candidates based on the KATZ measurement (Zhou et al., 2020). Figure 2 shows the AUC values computed by these four CDA prediction methods.

From Figure 2, we can find that CDA-EnRWLRLS is significantly better than CD-LNLP (Zhang et al., 2019), DWNN-RLS (Yan et al., 2018), and KATZHCDA (Fan et al., 2018b) based on the AUC value. Compared to the three models, CDA-EnRWLRLS obtains the highest AUC of 0.8654, outperforming 7.60%, 24.86%, and 0.25%, respectively. In particular, DWNN-RLS used regularized least squares with Kronecker product kernel for CDA prediction. Disease similarity was computed by their semantic similarity and Gaussian association profile similarity. Meanwhile, circRNA similarity was computed by their Gaussian association profiles. CDA-EnRWLRLS uses an ensemble model to identify possible CDAs. Similar to DWNN-RLS, CDA-EnRWLRLS computes disease similarity. However, CDA-EnRWLRLS computes circRNA similarity by their functional similarity and Gaussian association profile similarity. Furthermore, CDA-EnRWLRLS still computes association score between each circRNA-disease pair using random walk with restart except Laplacian regularized least squares and integrates the results from the two models by the soft voting technique. Therefore, CDA-EnRWLRLS outperforms DWNN-RLS, which demonstrates its powerful CDA prediction ability.

### 3.3 Performance evaluation of ensemble learning model with individual models

**FIGURE 3:**
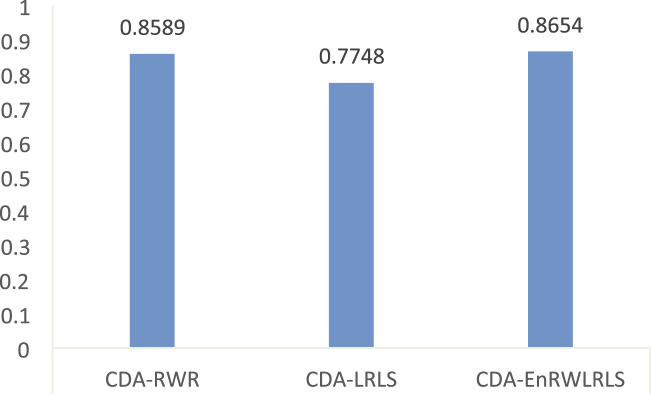
The AUC values of CDA-EnRWLRLS and CDA-RWR and CDA-LRLS.

Our proposed CDA-EnRWLRLS model is an ensemble of two state-of-the-art models (i.e., random walk with restart and Laplacian regularized least squares). To evaluate the performance of ensemble learning model and individual models, we conducted 5-fold cross validation experiment for CDA-EnRWLRLS and random walk with restart (CDA-RWR) and Laplacian regularized least squares (CDA-LRLS) on the CDA dataset. Figure 3 shows the AUC values computed by CDA-EnRWLRLS, CDA-RWR, and CDA-LRLS. From Figure 3, we can find that CDA-EnRWLRLS obtains better AUC than two individual models, CDA-RWR and CDA-LRLS, which shows that the proposed ensemble learning-based model can outperforms individual models.

### 3.4 Evaluation of parameter sensitivity

**FIGURE 4:**
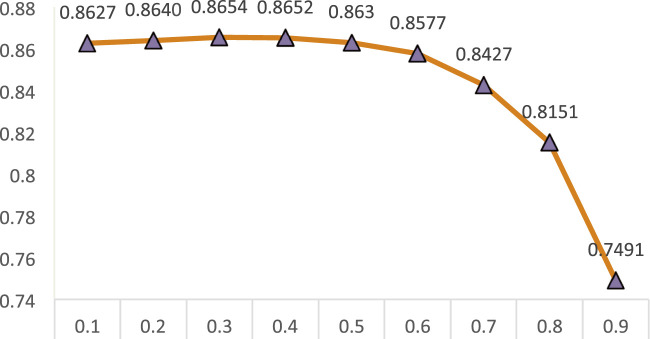
The effect of θ on the prediction performance for CDA-EnRWLRLS.

In this study, we ensemble two individual models, random walk with restart and Laplacian regularized least squares. However, the two models may have different effects on the CDA prediction performance. To evaluate their effect on the performance, we consider θ in the range of [0.1, 0.9] with stride of 0.1. The results are shown in Figure 4.

From Figure 4, we can find that AUC computed by CDA-EnRWLRLS gradually increases when the parameter θ is from 0.1 to 0.3. Its computed AUCs gradually decrease when the parameter θ is from 0.3 to 0.9. In other words, CDA-EnRWLRLS obtains the best AUC when the parameter θ is 0.3. Thus, the parameter θ is finally set as 0.3.

### 3.5 Case study

We consequently compute the association score for each circRNA-disease pair. In particular, we compute association abilities between all circRNAs and bladder cancer and bladder urothelial cancer to analyze any possible associations between these circRNAs and the two cancers, and to further screen for potential circRNA biomarkers for them.

#### 3.5.1 circRNA biomarker analysis for bladder cancer

Bladder cancer is a heterogeneous disease with high morbidity and mortality rates (Kamat et al., 2016). It has been estimated that about 73,510 new cases of bladder cancer were diagnosed in the United States in 2012. During the same period, about 14,880 patients died from bladder cancer (Clark et al., 2013). To analyze circRNA biomarkers for bladder cancer, we compute association between all circRNAs and bladder cancer after training CDA-EnRWLRLS. Table 1 gives the top 20 circRNAs that are predicted to have the highest association scores with bladder cancer.

**TABLE 1:** The inferred top 30 circRNAs associated with bladder cancer.

Rank	circRNAs	Evidence
1	hsa_circ_0000172	circRNADisease
2	hsa_circ_0002495	circRNADisease
3	Chr22: 28943661	circRNADisease
4	Chr5: 158368701	circRNADisease
5	Chr9: 74522734	circRNADisease
6	circRNA BCRC4/hsa_circ_001598/hsa_circ_0001577	circRNADisease
7	hsa_circ_0003221/circPTK2	circRNADisease
8	hsa_circ_0091017	circRNADisease
9	hsa_circ_0002024	circRNADisease
10	circMylk/circRNA-MYLK/hsa_circ_0002768	circRNADisease
11	circTCF25/hsa_circ_0041103	circRNADisease
12	circFAM169A/hsa_circ_0007158	circRNADisease
13	circTRIM24/hsa_circ_0082582	circRNADisease
14	circBC048201/hsa_circ_0061265	circRNADisease
15	hsa_circRNA_100782/circHIPK3/hsa_circ_0000284	Unconfirmed
16	circZFR/hsa_circRNA_103809/hsa_circ_0072088	Unconfirmed
17	Cir-ITCH/hsa_circ_0001141/hsa_circ_001763	Unconfirmed
18	circSMARCA5/hsa_circ_0001445	PMID: 35712125, 35116915, 34482767
19	hsa_circ_0001649	PMID: 35200157
20	CDR1as/ciRS-7/hsa_circ_0001946	PMID: 29694981, 31131537, 33335899

**FIGURE 5:**
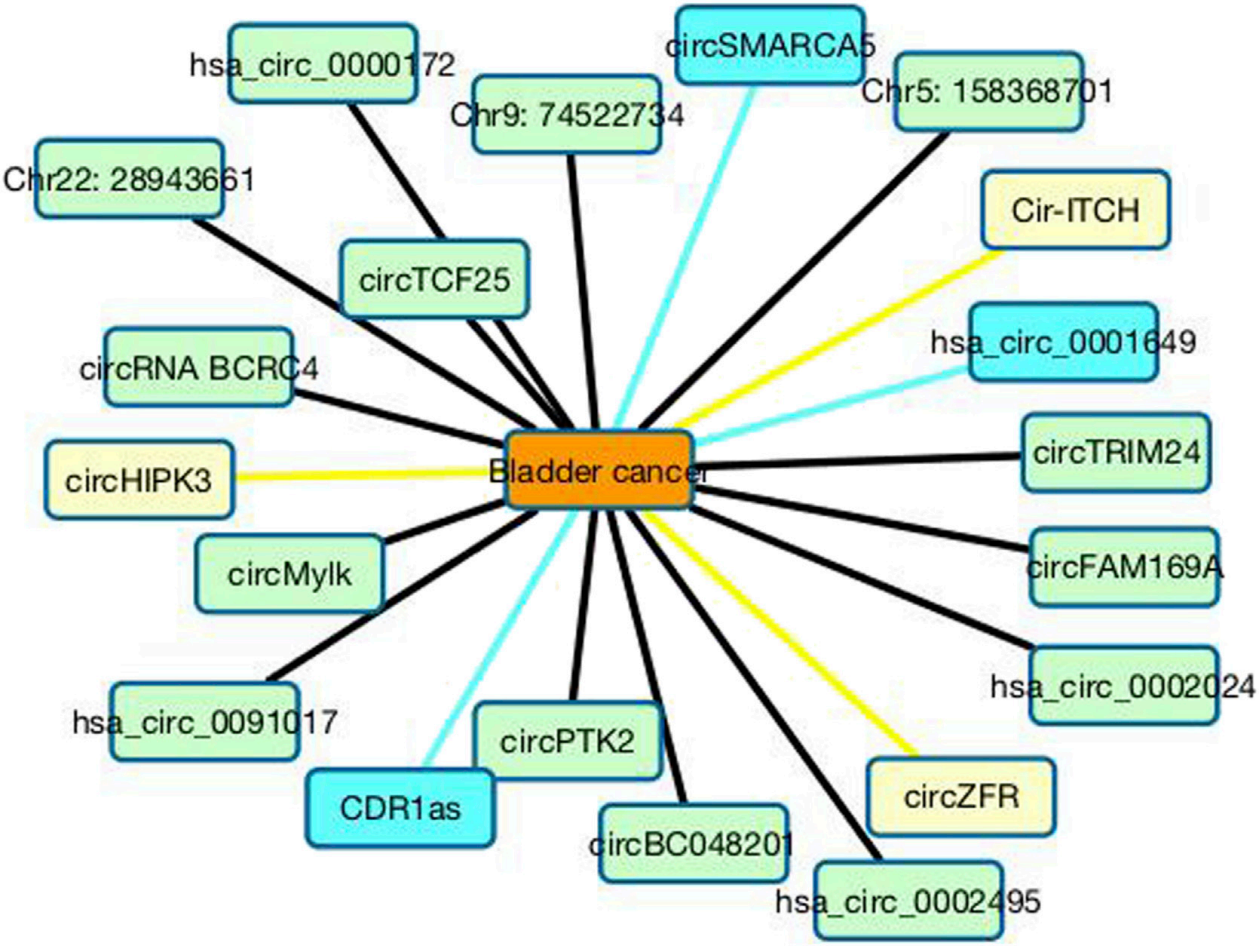
Associations between the top 20 circRNAs with bladder cancer. Black lines represent associations that have validated in the CDA dataset. Sky blue lines represent associations that are unknown in the CDA dataset but can be validated by related literatures. Yellow lines represent association that are unknown in the CDA dataset and need validation.

In the CDA dataset, 15 circRNAs are known to associate with bladder cancer among 585 circRNAs. From Table 1, we can find that the 15 circRNAs are predicted to have the highest association scores with bladder cancer and are ranked as the top 15. Furthermore, we predict that circHIPK3 may associate with bladder cancer with the ranking of 16. Furthermore, circHIPK3 is a promising cancer-related circRNA (Zhang et al., 2020). It can regulate cell growth through sponging multiple miRNAs (Zheng et al., 2016). For instance, circHIPK3 can regulate cell proliferation and migration in hepatocellular cancer by sponging miR-124 (Chen X. et al., 2018), modulate autophagy in STK11 mutant lung cancer (Chen et al., 2020), and promote glioma progression as a prognostic marker (Jin et al., 2018). The overexpression of circHIPK3 can accelerate the proliferation and invasion of prostate cancer cells (Cai et al., 2019). Its inhibition can block angiotensin II-induced cardiac fibrosis (Ni et al., 2019). In this study, we infer that circHIPK3 may be a biomarker of bladder cancer and need experimental validation. Figure 5 shows the association information between the top 20 circRNAs with bladder cancer.

#### 3.5.2 circRNA biomarker analysis for bladder urothelial cancer

Over 90% bladder cancer is bladder urothelial cancer. Bladder urothelial cancer is a common malignancy with high morbidity and mortality worldwide (Cancer Genome Atlas Research Network, 2014). In the United Sates, bladder urothelial cancer is one of the main histologic subtypes (Clark et al., 2013). However, no molecularly targeted agent has been applied to the treatment, until now. To infer potential circRNA biomarkers for bladder urothelial cancer, we compute association scores between all circRNAs and bladder urothelial cancer using CDA-EnRWLRLS. Table 2 gives the top 20 circRNAs that are predicted to have the highest association scores with bladder urothelial cancer.

**TABLE 2:** The inferred top 30 circRNAs associated with bladder urothelial cancer.

Rank	circRNAs	Evidence
1	hsa_circRNA_100782/circHIPK3/hsa_circ_0000284	circRNADisease
2	circSMARCA5/hsa_circ_0001445	Unconfirmed
3	hsa_circ_0001649	Unconfirmed
4	Cir-ITCH/hsa_circ_0001141/hsa_circ_001763	Unconfirmed
5	CDR1as/ciRS-7/hsa_circ_0001946	PMID: 32658427
6	circZFR/hsa_circRNA_103809/hsa_circ_0072088	Unconfirmed
7	CircDOCK1/hsa_circ_100721	Unconfirmed
8	circRNA_100290/hsa_circ_0013339/hsa_circ_100290	Unconfirmed
9	circPVT1/hsa_circ_0001821	PMID: 34902986
10	hsa_circ_0001313/circCCDC66	Unconfirmed
11	circGFRA1/hsa_circ_005239	Unconfirmed
12	circZNF609/hsa_circ_0000615	Unconfirmed
13	circWDR77/hsa_circ_0013509	Unconfirmed
14	hsa_circ_0000096/circHIAT1/hsa_circ_001013	Unconfirmed
15	circRNA_000167/hsa_circRNA_000167/hsa_circ_0000518	Unconfirmed
16	hsa_circ_0007534	Unconfirmed
17	circPRKCI/hsa_circ_0067934	Unconfirmed
18	hsa_circRNA_103110/hsa_circ_103110/hsa_circ_0004771	Unconfirmed
19	circ-Foxo3/hsa_circ_0006404	PMID: 31903146
20	circFUT8/hsa_circRNA_101368/hsa_circ_0003028	Unconfirmed

**FIGURE 6:**
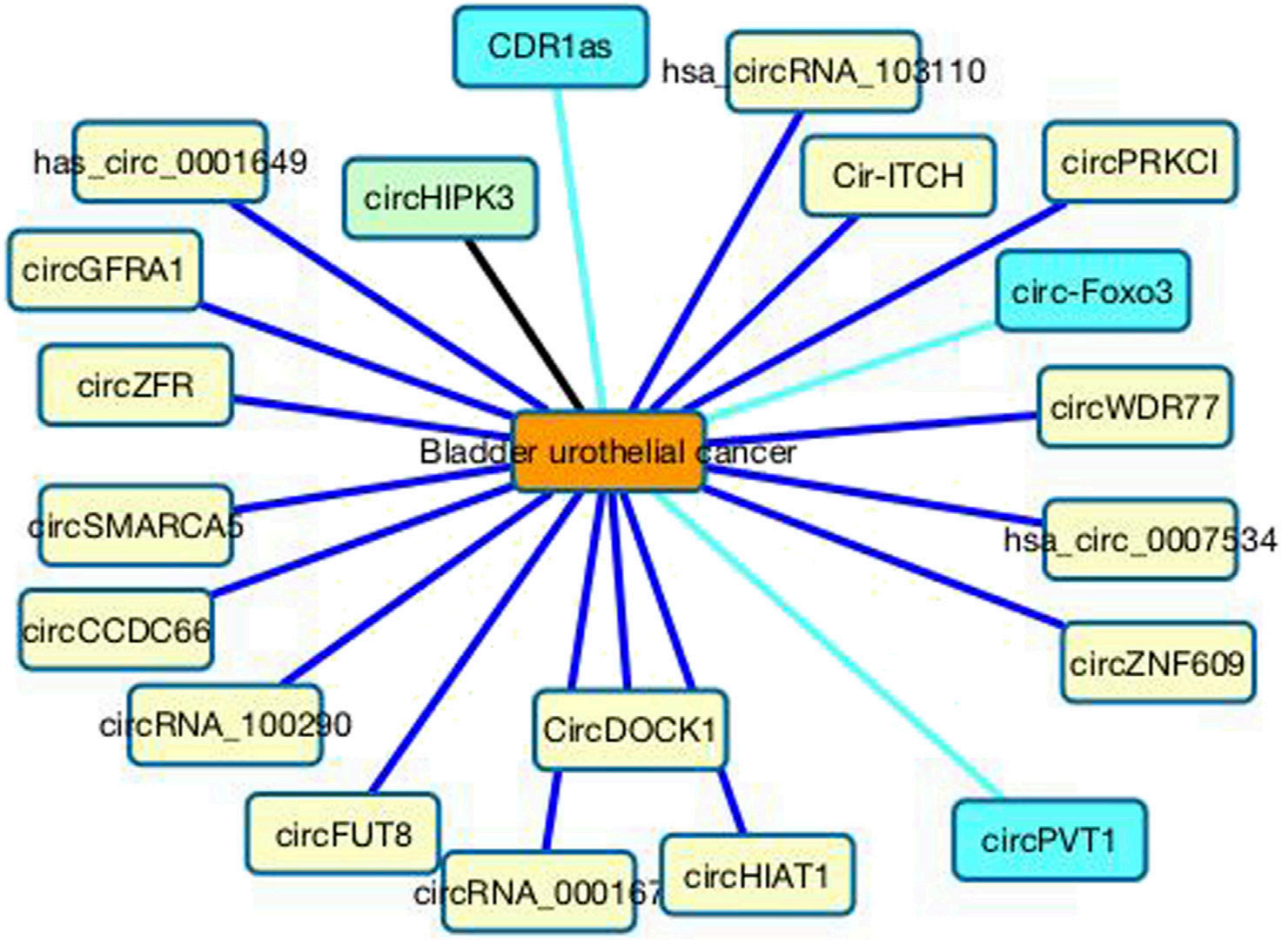
Associations between the top 20 circRNAs with bladder urothelial cancer. Black lines represent associations that have validated in the CDA dataset. Sky blue lines represent associations that are unknown in the CDA dataset but can be validated by related literatures. Blue lines represent association that are unknown in the CDA dataset and need validation.

In the CDA dataset, only one circRNA, circHIPK3, associates with bladder urothelial cancer among all potential 585 circRNAs. We predict that SMARCA5 may associate with bladder urothelial cancer with the ranking of 2. SMARCA5 is a member of the ISWI family that is involved in chromatin remodeling. It can regulate chromosome remodeling through diverse mechanisms, hinder cell proliferation, and assist apoptosis by sponging miRNAs. Its expression may boost the susceptibility of cells to chemotherapy, boost the sensitivity of cancer detection, promote early diagnosis, and help the treatment of chemotherapy-resistant cancers (Qin and Wan, 2022). Its expression level has a certain association with clinical features of many cancers. For instance, SMARCA5 can promote cell proliferation in bladder cancer and prostate cancer (Tan et al., 2019), suppress colorectal cancer progression (Miao et al., 2020), inhibit tumor metastasis in cervical cancer (Zhang X. et al., 2021) and inhibit cell proliferation, migration, and invasion in non-small cell lung cancer (Wang et al., 2019), and boost cell migration and invasion as well as inhibit cell apoptosis in bladder cancer (Kong et al., 2017; Tan et al., 2019). Many studies have reported that circSMARCA5 plays a key role in the occurrence and development of cancer. Moreover, it also serves as a reliable indicator of tumor screening or cancer prognosis evaluation (Qin and Wan, 2022). Therefore, SMARCA5 is a diagnostic and prognostic biomarker of cancer and has obtained wide attention. In this study, we predict that SMARCA5 may be potential biomarker of bladder urothelial cancer; however, this needs validation. Figure 6 shows the association information between the top 20 circRNAs with bladder urothelial cancer.

## 4 Discussion and conclusion

Bladder cancer, including bladder urothelial cancer, is a common and complex disease. These cancers have caused high morbidity and mortality. The identification of biomarkers for bladder cancer and bladder urothelial cancer can help in their prognosis and treatment. In this manuscript, we developed an ensemble learning model, CDA-EnRWLRLS, to discover potential circRNA biomarkers for the two cancers based on CDA association prediction.

CDA-EnRWLRLS first computes circRNA similarity and disease similarity by fusing semantic similarity and association profile similarity of diseases and functional similarity and association profile similarity of circRNAs. Second, it scores each circRNA-disease pair by random walk with restart and Laplacian regularized least squares, respectively. Third, the results computed by random walk with restart and Laplacian regularized least squares are integrated by the soft voting approach based on ensemble learning. Finally, it is applied to discover potential circRNA biomarkers for bladder cancer and bladder urothelial cancer.

CDA-EnRWLRLS is compared to three classical CDA prediction methods (CD-LNLP, DWNN-RLS, and KATZHCDA) and two individual models (CDA-RWR and CDA-LRLS). The results show that CDA-EnRWLRLS computes relatively better AUC, which demonstrates its relatively powerful CDA prediction ability. We predict that circHIPK3 and SMARCA5 may be potential biomarkers of bladder cancer and bladder urothelial cancer, respectively.

CDA-EnRWLRLS has two advantages: on the one hand, it better fuses biological features and association features of diseases and circRNAs; while on the other hand, it combines two individual classical association prediction models to obtain the powerful association prediction performance from different bioinformatics tools. Although CDA-EnRWLRLS computed better CDA inference ability, the circRNA functional similarity was calculated indirectly by disease semantic similarity. Moreover, its prediction performance needs further improvement. In the future, we will consider biological features of circRNAs and develop more efficient machine learning, especially ensemble learning models (Zhou et al., 2021a; Peng et al., 2022a) and deep learning models (Peng et al., 2021b; Zhou et al., 2021b; Sun et al., 2022; Yang et al., 2022) to discover potential biomarkers for bladder cancer and bladder urothelial cancer.

## Data Availability

The original contributions presented in the study are included in the article/supplementary material, and further inquiries can be directed to the corresponding author.
